# The Effect of Polymer Elastic Particles Modified with Nano-Silica on the Mechanical Properties of Oil Well Cement-Based Composite Materials

**DOI:** 10.3390/polym15143130

**Published:** 2023-07-23

**Authors:** Xiaoliang Wang, Mingbiao Xu, Yi Qin, Jianjian Song, Rongyao Chen, Zhong Yin

**Affiliations:** 1 School of Petroleum Engineering, Yangtze University, Wuhan 430100, China; 2 National Engineering Research Center for Oil and Gas Drilling and Completion Technology (Yangtze University), Wuhan 430100, China; 3 Hubei Key Laboratory of Oil and Gas Drilling and Production Engineering (Yangtze University), Wuhan 430100, China; 4 No.1 Cementing Company, CNPC Bohai Drilling Engineering Company Limited, Renqiu 062552, China

**Keywords:** polymer particles, oil well cement, cementing, cement sheath, mechanical properties

## Abstract

The integrity of oil well cement sheaths is closely related to the long-term production safety of oil and gas wells. The primary material used to form a cement sheath is brittle. In order to reduce the brittleness of oil well cement and improve its flexibility and resistance to stress damage, nano-silica was used to modify polymer elastic particles, and their properties were analyzed. The influence of the modified polymer particles on the properties of oil well cement-based composite materials was studied, and the microstructure of the polymer particle cement sample was analyzed. The results showed that nano-silica effectively encapsulates polymer particles, improves their hydrophilicity, and achieves a maximum temperature resistance of 415 °C. The effect of the modified polymer particles on the compressive strength of cement sample is reduced. Polymer particles with different dosages can effectively reduce the elastic modulus of cement paste, improve the deformation and elasticity of cement paste, and enhance the toughness of cement paste. Microstructural analysis showed that the polymer particles are embedded in the hydration products, which is the main reason for the improvement in the elasticity of cement paste. At the same time, polymer particle cement slurry can ensure the integrity of the cement sample after it is impacted, which helps to improve the ability of oil well cement-based composite materials to resist stress damage underground.

## 1. Introduction

With the long-term production of oil and gas wells and the increasing complexity of exploration and development objects, there are an increasing number of accidents of oil and gas wells being shut down or scrapped due to wellbore failure, which makes the integrity management of oil- and gas-well-cementing barriers increasingly important [1,2]. Maintaining the integrity of the cementing barrier reduces the risk of oil and gas field development, thereby ensuring the safety of the entire production period of the oil and gas well. The primary functions of the cement sheath are to effectively protect the production casing string and seal the adjacent oil, gas, and water layers. If the cement sheath seal fails, it will cause pressure or flow in the annulus, and in severe cases, it will cause damage to the casing and even lead to the scrapping of the oil and gas well, resulting in severe economic losses [3,4,5,6]. Oil well cement is the main component of cementing materials. As oil well cement is a brittle material, it is prone to microcracks or micro-annuli during subsequent operations such as perforation, fracturing, and mining after cementing, as well as the complex forces of underground rock formations, which will damage the integrity of the cement sheath and affect the safe and efficient exploitation of oil and gas wells.

When the elastic modulus of an oil well cement-based composite material is relatively low, it can effectively ensure the integrity of the cement sheath under complex stress in the wellbore [7,8]. Polymer elastic particles have been proven to reduce the elastic modulus of cement-based materials [9,10,11]. After polymer particles are added to cement-based composites, they form a flexible structural center inside the cement matrix. When the cement slurry is compressed, this flexible structure will deform, improving the deformation ability and the elasticity of the cement slurry. However, all these polymer particles need to be hydrophilically modified, mainly due to the hydrophobic groups on the surface of the polymer particles, which have a lower interfacial adhesion force and lower co-crosslinking level. When applied to water-based mixed systems, this can cause a decrease in the stability, strength, toughness, and other physical properties of the mixed system [12,13,14,15]. Cheng et al. [16], He et al. [17], and Zhong et al. [18] found that unmodified rubber powder cannot be dispersed when soaked in water, and when added to cement-based composite materials, its compressive strength is low, which can easily lead to a poor stability of the cement paste and the deterioration of its mechanical properties. Therefore, the surface modification of polymer particles is a critical step in the application of polymer particles in cement-based composite materials.

At present, the main methods used for modifying polymer particles include plasma modification, silane coupling agent modification, and other methods [19,20,21]. To some extent, these methods can improve the hydrophilicity of polymer particles and enhance their dispersion ability in cement slurry. However, the surfaces of polymer particles modified using these methods still have a polymer structure, and the improvement effect of hydrophilicity is limited. Meanwhile, the research by Song et al. [22,23,24] showed that although polymer particles can improve the elasticity of cement paste, they can also have adverse effects on the compressive strength of cement paste. The more polymer particles are added, the more significant the decrease in the compressive strength of the cement paste will be. The current modification methods can only be used to modify polymer particles, which cannot overcome the negative impact of polymers on the compressive strength of cement paste [25].

Inorganic coating technology has been proven to be a useful polymer modification technology in recent years. Li et al. [26] investigated the influence of silica-coated rubber on the performance of rubber mortars. A classic Stöber sol–gel method is applied to produce a layer of silica coating on rubber particles. The silica coating on the rubber particles reduces the hydrophobicity of the rubber particles. Compared to unmodified rubber, the compressive strength and flexural strength of cement samples have been improved. In order to improve the bonding with the cementation matrix, Santos et al. [27] and Lee et al. [28] used the sol–gel method to prepare the surface of the polymer fiber. Compared with the unmodified fiber, the modified fiber had better mechanical properties. These studies indicate that coating inorganic materials onto the surfaces of polymers can improve their performance. However, the existing studies used the silica sol method for their modification experiments, which combines silicon elements with polymer materials to achieve modification effects. Due to the small size of polymer particles, if the silica powder coating is not thorough, the surface of the polymer particles will still exhibit hydrophobic properties. In particular, in oil and gas wells, which are often accompanied by high-temperature and high-pressure environments, if the coating treatment is not thorough, this will not only render the polymer particles unable to play their role but may also have negative effects.

In view of this issue, we used a proven effective silane coupling agent to treat the surfaces of polymer particles, improving their hydrophilicity. Subsequently, nano-silica was used to wrap the surfaces of rubber particles, further improving the dispersion of the polymer particles. The main advantage is that even if the nano-silica does not completely encapsulate the polymer particles, its hydrophilicity will still be improved. At the same time, the encapsulation of nano-silica further improves the hydrophilicity of polymer particles and allow them to participate in the hydration reaction of oil well cement, alleviating the adverse effect of the polymer particles on the mechanical properties of the cement paste [29,30,31]. This article characterizes the properties of nano-silica-modified polymer particles and studies the effect of modified polymer particles on the mechanical properties of oil well cement. These research results provide technical support for the design of polymer particle elastic cement slurry systems.

## 2. Experiment

### 2.1. Experimental Materials

G-grade oil well cement was purchased from the Gezhouba Special Cement Plant (China) as the primary cementitious material. The unmodified polymer particles were obtained from Jingzhou Jiahua Technology Co., Ltd. (Jingzhou, China). These polymers are styrene polymers and provide a flexible structural center in oil well cement-based composite materials. Due to the poor hydrophilicity of polymer particles, they need to be modified. The silane coupling agent 3-methyloxypropyltrimethoxysilane was used for the preliminary modification of polymer surfaces and was purchased from Jiangxi Cheng New Materials Co., Ltd., China (Jiujiang, China). Nano-silica is used to coat the surface of polymer particles and was purchased from Jingzhou Jiahua Technology Co., Ltd. The dispersant, filtrate reducer, and retarder were prepared in the laboratory. The main components of the dispersant and filtrate reducer were aldehyde ketone condensates and AMPS polymers, mainly used to improve the rheological properties and reduce the water loss of the cement slurry. At the same time, the fluid loss reducer could also improve the stability of cement slurry. The main component of the retarder was boric acid, which was used to regulate the thickening time of the oil well cement.

### 2.2. Experimental Methods

#### 2.2.1. Polymer Particle Modification Methods

A total of 50 g of unmodified polymer particles was weighed and wholly immersed in a 1% silane coupling agent solution. The silane coupling agent solution was stirred at 50 °C and 200 r/min to thoroughly disperse the polymer particles in the solution. The polymer particle solution was filtered and dried at 50 °C to obtain a rubber powder modified with a silane coupling agent. Subsequently, the polymer particles modified with the silane coupling agent were added to a 10% concentration nano-silica aqueous solution to evenly disperse the polymer particles in the nano-silica aqueous solution. The mixture was stirred at a speed of 100 r/min for 2 h, with a mass ratio of 10:1 between the polymer powder and nano-silica aqueous solution. Finally, we dried the polymer particles/nano-silica aqueous solution to obtain nano-silica-modified polymer particles.

#### 2.2.2. Analysis Method of the Modified Polymer Particles

Next, 2 mg of modified and unmodified polymer particles was weighed, mixed evenly with 200 mg of pure potassium bromide, and then pressed into a thin sheet. Infrared spectroscopy (Nicolet iS5, Thermo Fisher Scientific, Waltham, MA, USA) was used to analyze the functional groups on the surface of the sample.

A thermogravimetric analyzer (STA449F5, Netzsch, Selb, Germany) was used to evaluate the thermal stability of the polymer particles. The tested gas was nitrogen. The heating rate was 10 °C/min.

#### 2.2.3. Preparation of Cement Slurry

We weighed the cement, polymer particles, dispersant, and fluid loss additive in the specified proportions and mixed them evenly as a dry powder. We used a mixing cup to weigh the specified proportion of water and placed the mixing cup on a constant-speed mixer (TG-3060A, Shenyang Taige Petroleum Instrument Equipment Co., Ltd., Shenyang, China) for stirring at a speed of 4000 r/min, pouring the dry powder into a mixing cup to mix the cement slurry evenly. A cement slurry system was prepared. The composition of modified polymer particle cement slurries with different formulas is shown in Table 1.

#### 2.2.4. Evaluation of Mechanical Properties of Cement Sample

After the preparation of the cement slurry, the cement samples for stress–strain and impact strength testing were maintained at 60 °C/21 MPa. The stress–strain curve was tested using a universal mechanical testing machine (HY-20080, Shanghai Hengyi Precision Instrument Co., Ltd., Shanghai, China) at a constant compression speed of 0.4 kN/s, and the size of the cement sample was 50.8 mm × 50.8 mm × 50.8 mm in the shape of a cube. The impact strength was tested using an impact strength testing machine (XJJY-50, Chengde Shipeng Testing Equipment Co., Ltd., Chengde, China) with a sample size of 120 mm × 15 mm × 10 mm. Three samples were tested, and the average value was taken. All the mechanical properties were tested at room temperature and pressure.

#### 2.2.5. Microstructure of Cement Sample

After preparing the cement sample, we took small flat pieces from inside the cement stone for surface gold plating. The micromorphology and characteristics of the pure cement slurry and polymer-particle-modified cement stone were observed via scanning electron microscopy (SU8010, Hitachi, Tokyo, Japan) in the high-vacuum mode.

We prepared simulated perforation samples based on the cement slurry formula and conducted perforation tests. The sample to be perforated was a cylinder with a diameter of 100 mm and a thickness of 26 mm. We performed a CT scanning analysis of the simulated perforation samples using an industrial CT scanner (GE Vtomex, General Electric Company, Boston, MA, USA).

## 3. Results and Discussion

### 3.1. Characterization and Performance of Modified Polymer Particles

#### 3.1.1. Infrared Spectroscopy

**Figure 1:**
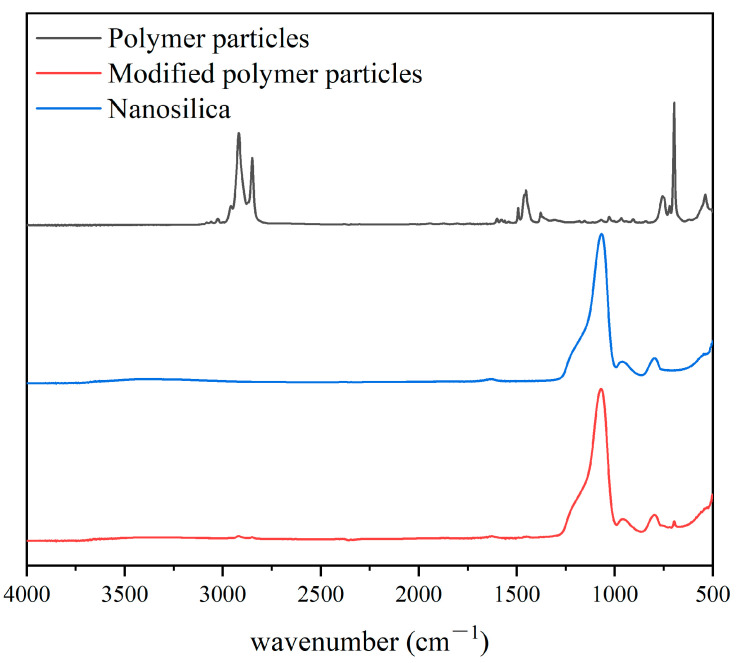
FT-IR spectra of sample.

Nano-silica was used to modify polymer particles. In order to analyze the functional group changes on the surface of polymer particles before and after modification, an infrared spectrometer was used to analyze the sample. The experimental results are shown in Figure 1. The experimental results indicate that there are more characteristic peaks for the unmodified polymer particles. There is a peak in the sample at 3024.88 cm^−1^, indicating that the sample contains C-H bond tensile vibrations on the benzene ring. At 2917.88 cm^−1^ and 2849.61 cm^−1^, the wave numbers correspond to =CH_2_. There is a characteristic peak of the benzene ring at 1452.65 cm^−1^. The wave numbers 755.84 cm^−1^ and 696.69 cm^−1^ correspond to the characteristic peaks of the monosubstituted benzene ring. The analysis results indicate that the sample contains hydrophobic groups such as benzene rings, resulting in hydrophobic polymer particles that cannot be well dispersed in water. For the nano-silica, its characteristic peaks are mainly at the wave numbers of 1065.85 cm^−1^, 960.38 cm^−1^ and 796.54 cm^−1^. This indicates the presence of Si-O in the sample, which is a distinct feature of silica. There are no hydrophobic groups on the surface of the nano-silica, which can be well dispersed in aqueous solutions. When the polymer particles are modified, their surface was completely enveloped by the nano-silica, and their main characteristic peaks are similar to those of nano-silica, indicating that the modified polymer particles bind well with nano-silica without any exposed surface and can be well dispersed in aqueous solutions.

#### 3.1.2. Thermogravimetric Analysis

**Figure 2:**
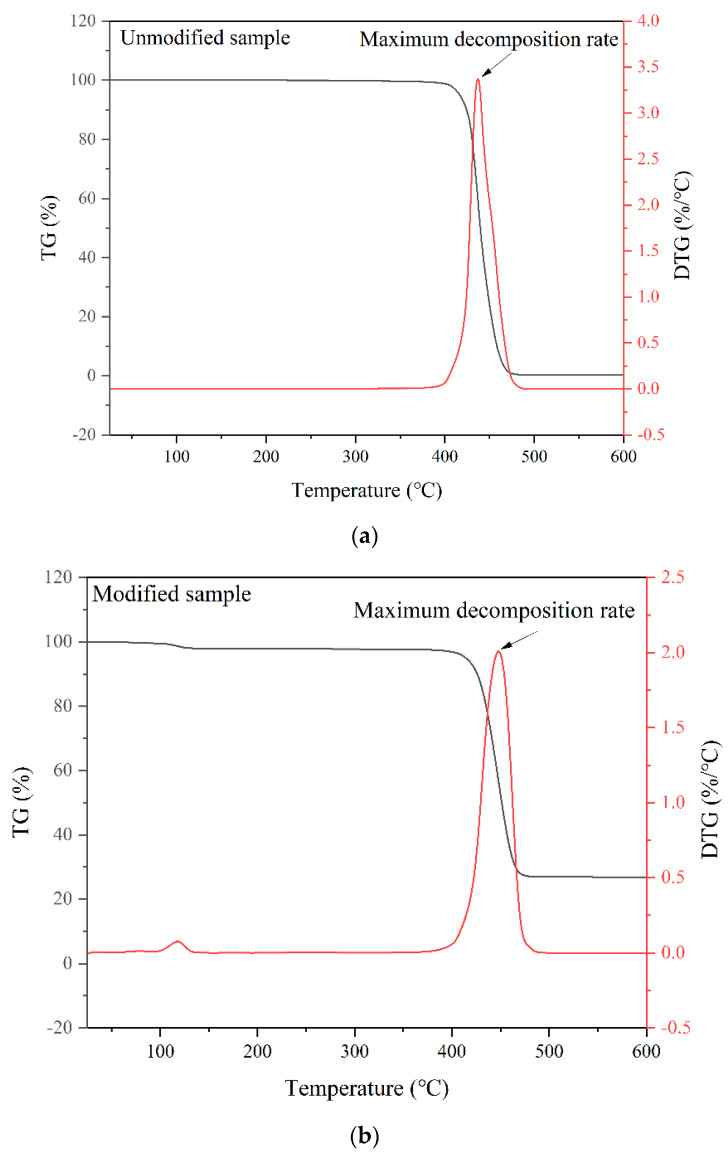
TG–DTG curve of the polymer particles ((**a**) unmodified sample; (**b**) modified sample).

Due to the fact that oil well cement slurry usually needs to be used at a particular temperature, there is a requirement for the thermal stability of polymer particles. The thermal stability of polymer particles is directly related to their applicability. The TG curves of the polymer particles before and after modification are shown in Figure 2.

Figure 2 shows the thermogravimetric analysis results of the modified and unmodified polymer particles. The mass loss of the different samples increases with the increase in temperature. For the unmodified polymer particles, the sample begins to decompose when the temperature reaches 415 °C, resulting in a mass loss of 5% and a decomposition rate of 0.42%/°C. As the temperature continues to rise, the weight loss of the sample reaches stability at 472 °C, and the remaining mass of the sample is 0.36%. For the modified polymer particles, the temperature at which the sample loses 5% of its weight is also approximately 415 °C, and the decomposition rate is 0.26%/°C. As the temperature continues to rise, the weight loss of the sample reaches stability at 472 °C, and the remaining mass of the sample is 27.6%. Comparing the thermal weight loss results of the two samples, it was found that the modification did not have a negative impact on the thermal stability of the polymer particles. The maximum decomposition rate of the unmodified polymer particles is 3.37%/°C, while the maximum decomposition rate of the modified polymer particles decreases to 2%/°C. These results indicate that the decomposition and maximum decomposition rate of the sample after modification with nano-silica decreases and the thermal stability of the modified polymer particles are excellent. The usage temperature is higher than the bottom hole temperature of oil and gas wells and can thus be applied in the flexible modification of oil well cement.

#### 3.1.3. Surface Contact Angle Analysis

**Figure 3:**
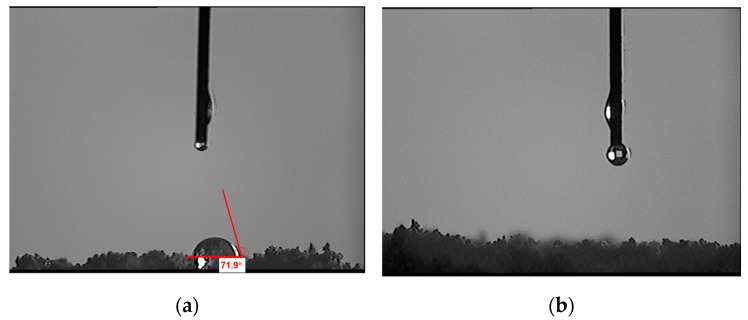
Surface contact angle of polymer particles before and after modification ((**a**) unmodified polymer particles, (**b**) modified polymer particles).

The contact angles of the different specimens were determined, as shown in Figure 3. From Figure 3, it can be seen that for the unmodified polymer particles, when water droplets are on their surface, their contact angle is relatively large, indicating that the polymer is not hydrophilic. When the polymer particles are modified, the contact angle cannot be observed after the water droplets are on the surface of the sample, indicating that the water is completely spread over the surface of the sample and the modified polymer particles have good hydrophilicity. The results of the analysis show that the hydrophilicity of polymer particles was greatly improved after modification.

#### 3.1.4. Comparison of the Compressive Strength of Oil Well Cement Slurry before and after Modification

**Figure 4:**
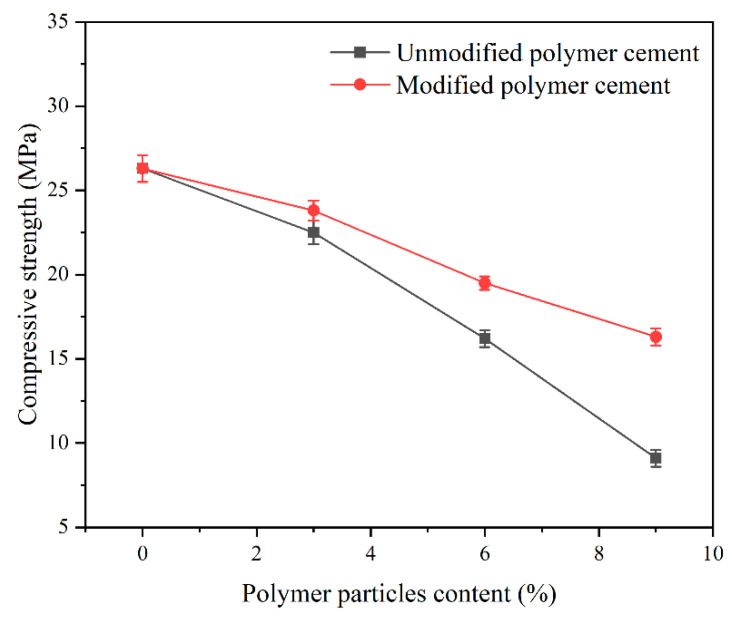
Comparison of the compressive strength of a polymer particle cement sample before and after modification.

The primary purpose of applying polymer particles to oil well cement is to form a flexible cement slurry system and improve the flexibility performance of cementing cement. Therefore, the application effect of polymer particles in cementing cement slurry before and after modification was evaluated. The compressive strength is the maximum external force that the cement stone can withstand when it is damaged; the cement stone needs to withstand the horizontal compressive force of the formation pressure and the vertical tension caused by the casing [32,33]. A cement sample needs to have a good compressive strength to support and protect the casing. The experimental results of the evaluation are shown in Figure 4.

From the experimental results in Figure 4, it can be seen that with the increase in the polymer particle content, the compressive strength of the cement slurry decreases. For the unmodified elastic particles, the compressive strength of the cement stone decreases significantly, mainly due to the non-hydrophilicity of the polymer powder, which prevents the elastic particles from mixing evenly with the cement slurry, resulting in the accumulation of elastic particles and the formation of significant defects. When elastic particles are modified, their hydrophilicity is improved, and they can be evenly mixed with cement slurry. On the one hand, the filling of flexible particles reduces the compressive strength of the cement paste, and on the other hand, the nano-silica used for modification can promote the hydration of cement slurry. The comprehensive effect of the modified elastic particles causes a slight decrease in the compressive strength of the cement slurry.

### 3.2. Mechanical Properties of Modified Flexible Particle Cement Sample

#### 3.2.1. Stress–strain Behavior of Cement Paste

In order to achieve a better long-term sealing quality, oil well cement-based composite materials need to have a greater flexibility and deformation ability. The stress–strain behavior is the deformation law of cement stone under external stress, which can be used to obtain the compressive strength and elastic modulus of cement stone. The elastic modulus can be regarded as an indicator with which to measure the difficulty of achieving elastic deformation in cement stone. The larger the value is, the smaller the elastic deformation and the greater the brittleness of the cement sample under a particular stress will be [34,35,36].

**Figure 5:**
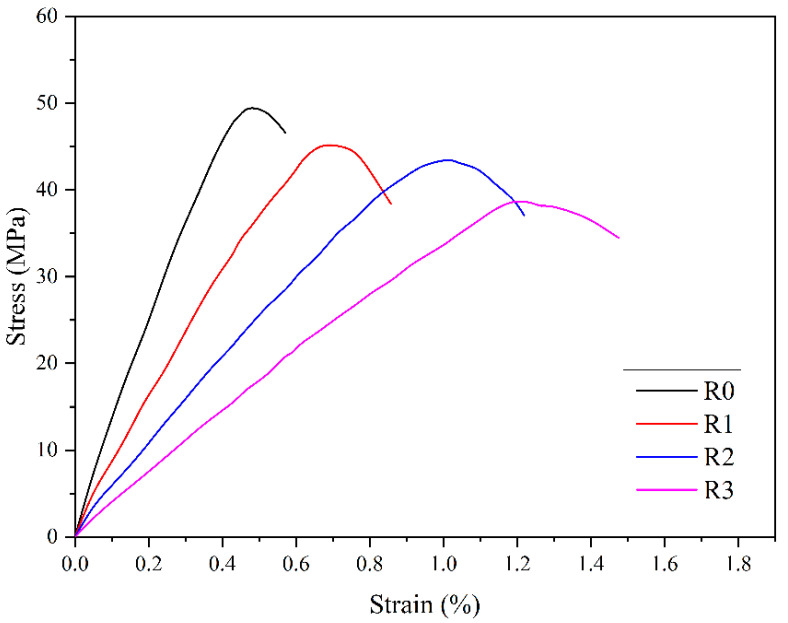
Stress–strain curves of cement samples.

The curve in Figure 5 indicates that under the external load, there is a significant difference in the stress–strain behavior of the cement paste before and after the addition of elastic particles. Under the same load, the shape of the cement paste with elastic particles changes significantly. When the cement stone is compressed, the strain increases with the increase in stress. When the sample reaches its maximum stress, the integrity of the cement stone is destroyed, and the stress decreases. However, the rate of stress decrease varies between different samples. After being destroyed, the stress of the pure cement sample rapidly decreases, while the stress of the sample containing elastic particles slowly decreases. This is because during the initial stage of external compressive stress, the internal pores of the cement stone are gradually compacted. As the load continues to increase, the cement stone enters an unstable state. When the maximum stress that the cement stone can withstand is reached, the cement stone is destroyed, and the destruction of the cement stone is a gradual process [37]. A cement sample with good flexibility still has a specific bearing capacity after the stress reaches its peak, so that the stress decreases slowly.

From the results in Table 2, it can be seen that compared with the pure cement paste, the maximum strain of sample R3 increased by 153.2%, and the elastic modulus decreased by 61.3%. The experimental results show that elastic particles significantly improve the deformation ability of cement paste and reduce its elastic modulus. This is of great significance for enhancing the ability of cement stone to resist well load damage.

#### 3.2.2. Impact Strength of Cement Paste

**Figure 6:**
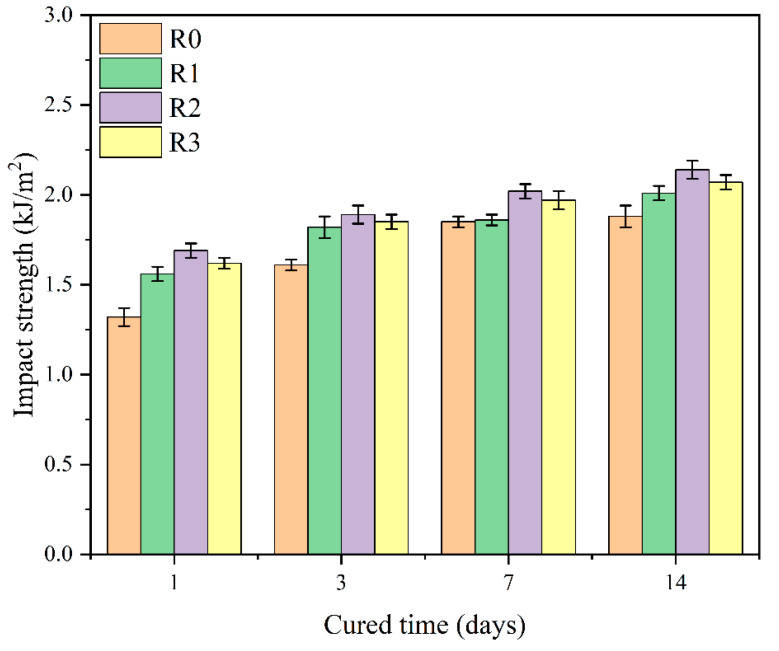
Impact strength of cement samples.

Impact strength is an essential indicator for testing the toughness of cement paste, and its size can directly reflect the toughness of cement paste and quantitatively characterize its toughening effect [38,39]. The impact strength of the polymer particle cement sample was evaluated, and the experimental results are shown in Figure 6. It can be seen that the impact strength of the flexible cement samples is higher than that of the pure cement at different curing ages. With the extension of the curing time, the increase in the impact strength of the cement paste is greater with the addition of more polymer particles. When the curing period is 14 days, the impact strength of sample R2 is the highest. When the curing time is 14 days, the impact strengths of the polymer particle cement samples R1, R2, and R3 are 6.9%, 13.8%, and 10.1% higher than the value of the pure cement sample R0, respectively. The experimental results indicate that the addition of polymer particles is beneficial for the impact strength of cement paste, which can enhance its toughness and enhance its ability to resist external impacts.

### 3.3. Microstructure of Modified Polymer Particle Oil Well Cement

#### 3.3.1. Scanning Electron Microscopy

**Figure 7:**
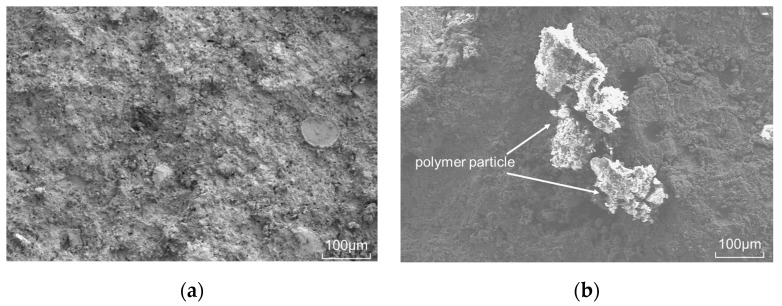
Microscopic morphology of modified polymer particle cement ((**a**) cement sample without polymer particles; (**b**) modified polymer particle cement sample).

From Figure 7, it can be seen that particle filling cannot be observed in the cement samples without polymer particles, and the microstructure of the cement paste is mainly composed of the cement matrix. In the cement samples containing polymer particles, it can be observed that polymer particles fill in the oil well cement stone and are bound to the oil well cement matrix. Due to the fact that polymer particles are connected by a large number of flexible chain segments, when elastic particles with an ultra-low elastic modulus are “embedded” in the cement paste, the particles fill in the spaces between the cement hydration products, forming a flexible structure with elastic particles as the core inside the cement paste. When the cement paste is compressed, the compressive stress is transferred from the external cement matrix to the elastic particles. The elastic particles form a structural deformation center within the cement paste matrix, which can limit the generation and development of strain microcracks, absorb strain energy, and improve the elasticity of the cement paste [40,41,42]. At the same time, when the cement sample is impacted by external loads, the crystal particles are the transmission medium of the impact force. The external impact load is transmitted through the cement matrix to the elastic particles filled in the middle of the cement stone matrix, buffering the impact force and absorbing some energy, thereby improving the impact resistance of the cement sample.

#### 3.3.2. CT Scanning of Simulated Perforated Specimens

**Figure 8:**
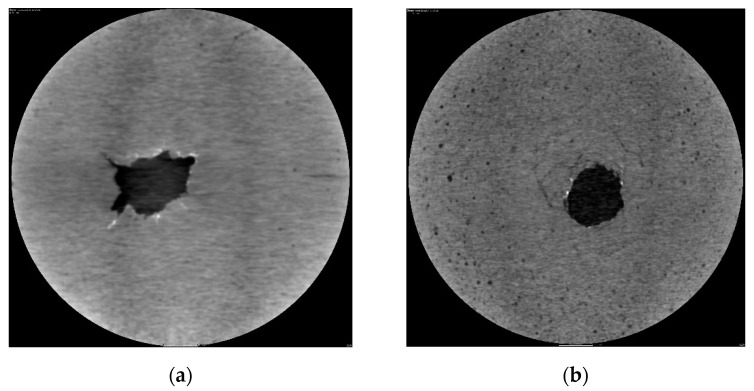
CT scanning micrograph of simulated perforated sample ((**a**) conventional cement sample; (**b**) modified polymer particle cement sample).

In order to study the ability of cement stone to retain its integrity after being subjected to downhole perforation operations, a CT scanner was used to analyze the structures of the different samples after perforation. The experimental results are shown in Figure 8. From Figure 8, it can be seen that there were many irregular cracks around the hole after the conventional cement sample was subjected to perforation impact. This is mainly because of its poor ability to resist impact loads. When the cement matrix around the hole was subjected to concentrated stress, it could not resist the impact, and it showed more crack propagation, which is not conducive to the resistance of a cement sample to underground stress. For a flexible cement paste, when subjected to stress impact, the pores formed by the oil well cement are regular, without apparent cracks, and have strong resistance to impact loads, which is conducive to ensuring the integrity of the long-term sealing of the cement sample underground. The evaluation results indicate that the flexible cement sample with modified elastic particles has strong resistance to underground stress damage.

## 4. Conclusions

This paper evaluated the performance of nano-silica modified polymer elastic particles and their impact on the performance of cement slurry. The modified polymer elastic particles have no hydrophobic groups on their surfaces, which helps them to disperse in an aqueous solution and have a good thermal stability. The compressive strength of the prepared cement slurry was higher than that of that unmodified elastic particle cement paste. After adding modified polymer elastic particles to the cement slurry, the compressive strength of the cement paste decreased, while the elastic modulus significantly decreased, the deformation greatly increased, and the impact strength increased. The polymer elastic particles were uniformly dispersed inside the cement stone, forming a flexible structural center that significantly improved the elastic toughness of the cement stone. After the perforation simulation, there were no micro-cracks formed in the polymer particle cement paste, and the pores were complete, indicating that the cement paste prepared using polymer elastic particles had a good flexibility. The oil well cement slurry prepared with this modified polymer particle could replace conventional oil well cement slurry for complex cementing operations such as those involving shale gas wells and tight oil gas wells, which will lead to better economic benefits.

## Figures and Tables

**Table 1:** Slurry components of different modified polymer particle formulas (wt.%).

Sample Number	Cement	Water	Filtrate Reducer	Dispersant	Retarder	Modified Polymer Particles
R0	100	44	2	0.8	0.4	0
R1	100	44	2	0.8	0.4	3
R2	100	44	2	0.8	0.4	6
R3	100	44	2	0.8	0.4	9

**Table 2:** Compressive properties of the samples.

Sample Number	Compressive Strength (MPa)	Maximum Strain (%)	Elastic Modulus (GPa)
R0	49.6	0.47	10.6
R1	45.3	0.67	7.8
R2	43.5	1.01	5.3
R3	38.7	1.19	4.1

## Data Availability

The data are contained within the article.
